# Review of Detection Limits for Various Techniques for Bacterial Detection in Food Samples

**DOI:** 10.3390/nano14100855

**Published:** 2024-05-14

**Authors:** Xinyi Zhao, Abhijnan Bhat, Christine O’Connor, James Curtin, Baljit Singh, Furong Tian

**Affiliations:** 1 School of Food Science and Environmental Health, Technological University Dublin, Grangegorman, D07 ADY7 Dublin, Ireland; d20127084@mytudublin.ie (X.Z.); d21126441@mytudublin.ie (A.B.); christine.oconnor@tudublin.ie (C.O.); baljit.singh@tudublin.ie (B.S.); 2 FOCAS Research Institute, Technological University Dublin, Camden Row, D08 CKP1 Dublin, Ireland; 3 MiCRA Biodiagnostics Technology Gateway and Health, Engineering & Materials Sciences (HEMS) Research Hub, Technological University Dublin, D24 FKT9 Dublin, Ireland; 4 Faculty of Engineering and Built Environment, Technological University Dublin, Bolton Street, D01 K822 Dublin, Ireland; james.curtin@tudublin.ie

**Keywords:** limit of detection (LOD), bacterial detection, LFIA, PCR, electrochemical method

## Abstract

Foodborne illnesses can be infectious and dangerous, and most of them are caused by bacteria. Some common food-related bacteria species exist widely in nature and pose a serious threat to both humans and animals; they can cause poisoning, diseases, disabilities and even death. Rapid, reliable and cost-effective methods for bacterial detection are of paramount importance in food safety and environmental monitoring. Polymerase chain reaction (PCR), lateral flow immunochromatographic assay (LFIA) and electrochemical methods have been widely used in food safety and environmental monitoring. In this paper, the recent developments (2013–2023) covering PCR, LFIA and electrochemical methods for various bacterial species (*Salmonella*, *Listeria*, *Campylobacter*, *Staphylococcus aureus* (*S. aureus*) and *Escherichia coli* (*E. coli*)), considering different food sample types, analytical performances and the reported limit of detection (LOD), are discussed. It was found that the bacteria species and food sample type contributed significantly to the analytical performance and LOD. Detection via LFIA has a higher average LOD (24 CFU/mL) than detection via electrochemical methods (12 CFU/mL) and PCR (6 CFU/mL). *Salmonella* and *E. coli* in the Pseudomonadota domain usually have low LODs. LODs are usually lower for detection in fish and eggs. Gold and iron nanoparticles were the most studied in the reported articles for LFIA, and average LODs were 26 CFU/mL and 12 CFU/mL, respectively. The electrochemical method revealed that the average LOD was highest for cyclic voltammetry (CV) at 18 CFU/mL, followed by electrochemical impedance spectroscopy (EIS) at 12 CFU/mL and differential pulse voltammetry (DPV) at 8 CFU/mL. LOD usually decreases when the sample number increases until it remains unchanged. Exponential relations (R^2^ > 0.95) between LODs of *Listeria* in milk via LFIA and via the electrochemical method with sample numbers have been obtained. Finally, the review discusses challenges and future perspectives (including the role of nanomaterials/advanced materials) to improve analytical performance for bacterial detection.

## 1. Introduction

Foodborne illnesses can be dangerously infectious, and they are predominantly caused by pathogens (e.g., bacteria, fungi, viruses, parasites, etc.) or toxins (e.g., dioxins, heavy metals, mycotoxins, etc.) entering the body through contaminated food [1]. Most of the pathogens that can cause foodborne diseases are bacteria [2]. Bacteria can cause acute poisoning, long-term diseases, serious disabilities and even deaths [3]. Among all the bacteria species, *Salmonella* causes the most serious illnesses and deaths related to contaminated food [4,5]. *Salmonella* is commonly found in birds, vegetables and also in natural water [6]. Its symptoms include fever, vomiting, pain and dehydration, etc. [7]. *Salmonella* can be divided into over 2600 species. Among them, *Salmonella enterica* and *Salmonella typhimurium* are the most commonly found [8]. *Listeria* usually exists in processed products such as milk and meat and can grow in refrigerators [9]. *Listeria* is shown to cause miscarriages in pregnant women or deaths of infants, although the chance is low [10]. Around 20 species in *Listeria* can cause human diseases, and *Listeria monocytogenes* causes the most harm to humans [11]. Most *Campylobacter* infections in humans are acquired by eating and touching contaminated poultry and seafood [12]. More than 20 species of *Campylobacter* have been implicated in human disease, and the most well-known ones are *Campylobacter jejuni* and *Campylobacter coli* [13]. The most common symptoms of *Campylobacter* infections are diarrhea, fever, vomiting and stomach cramps [14]. *S. aureus* is normally found in birds, meat and milk [15]. *S. aureus* is one of the common bacteria species that display antimicrobial resistance to antibiotics like methicillin and vancomycin [16]. Common symptoms of *S. aureus* are shown on the skin, such as painful red welts and sores [17]. Generally, *E. coli* can be found in contaminated meat, milk and vegetables [18]. *E. coli* can be divided into three main groups—Enteropathogenic, Enteroinvasive and Enterohemorrhagic. A strain of Enterohemorrhagic *E. coli* is the most toxic variant [19]. Although *E. coli* does not cause any symptoms in most healthy humans, it can lead to diarrhea, vomiting and sometimes fever [20].

As many bacteria species currently pose a major threat to humans, a quick, accurate and cheap method to detect bacteria in the environment is essential, especially for food samples [21,22]. The traditional method to detect bacteria is through culturing of bacteria, which includes isolating the bacteria and monitoring the growth of the colonies [23]. During the culture process, the bacterial colonies are fixed and stained on a glass slide and confirmed using microscopy observation in order to identify different bacteria species. This process is usually very time-consuming and labor-intensive [24]. Other methods are more complex and can overcome some limitations of bacteria culture. Another common detection method is high-performance liquid chromatography (HPLC), which has high sensitivity [25,26]. When the concentration of bacterial colonies is very low but still cannot be ignored for human health, it poses a challenge for these methods [27]. Researchers have developed many alternative methods to overcome these problems [21]. One technology that has been widely used more recently is the enzyme-linked immunosorbent assay (ELISA), which is available as a commercial test kit for bacterial detection [28,29]. However, it has disadvantages, such as low sensitivity and very low temperature for storage [30]. As a result, it is very difficult to meet the demand for large-scale bacterial detection in food samples with current technologies.

PCR is a widely used method for bacterial detection in food [31]. It can make millions to billions of copies of a DNA sample rapidly, and LOD can be measured using copies of that DNA sample [32]. It has a high sensitivity and a relatively lower LOD than other common detection methods [33]. Commonly used nanomaterials in PCR are gold nanoparticles (AuNPs) and magnetic nanoparticles, which can speed up the PCR process and enhance its efficiency because they have good thermal conductivity [34]. LFIA, another widely used method for bacterial detection in food, is quick, cost-effective and simple to use [35]. LFIA usually provides qualitative and semi-quantitative but also quantitative results by measuring the color darkness of the test region in a strip [36]. It measures the concentration of bacteria using the darkness of color on the strip. Conjugated nanoparticles dominate the porous membrane as an indicator [37]. AuNPs and magnetic nanoparticles are the most-used nanoparticles in the LFIA because of their low toxicity, and particle size and shape can be controlled by many factors [8,38]. An alternative well-known method for bacterial detection in food is the electrochemical method [39,40]. This method mainly measures the changes in the electrochemical properties caused by bacteria introduced to the solution [41]. Carbon-based nanomaterial and metal nanoparticles are usually integrated onto the electrodes to capture bacteria efficiently and increase signal amplification [42].

Many reviews have been published on bacterial detection via different methods [43,44,45,46,47,48,49,50]. A review from Nnachi and co-authors compares nine methods for bacterial detection [43]. A review from Oh and co-authors discusses the influence of four pre-treatment methods for bacterial detection via PCR [44]. Furthermore, a review from Dey and co-authors discusses bacterial detection via LFIA in 10 bacteria species [45]. Moreover, a review from Vidic and co-authors discusses bacterial detection using an electrochemical method in eight sensor types [46]. However, they were not organized by a clear category (e.g., bacteria species, LOD, etc.) [43,44,45,46]. The LOD can be influenced by different conditions, and none of these articles has investigated how different conditions influence LOD systematically [47,48,49,50]. As a result, it is necessary to study the relationship between LOD and different conditions comprehensively. In this review, PCR, LFIA and electrochemical methods and their efficiency in the detection of different bacteria species in different food sample types have been summarized. Recent developments (2013–2023) cover PCR, LFIA and the electrochemical method for the detection of various bacterial species (*Salmonella*, *Listeria*, *Campylobacter*, *S. aureus* and *E. coli*) by considering the different food sample types, analytical performance and the reported LOD, as discussed from 150 peer-reviewed articles. Current challenges and future avenues to further improve analytical performance for bacterial detection are discussed.

## 2. Research Methods

Information was collected from Science Direct with these keywords: bacteria, PCR, LFIA, electrochemical method, LOD. A total of 150 peer-reviewed articles from 2013 to 2023 were compared to identify the LOD for different bacteria species—bacteria in the Pseudomonadota domain, which include *Salmonella* and *E. coli*; the Campylobacterota domain, which includes *Campylobacter*; and the Bacillota domain, including *Listeria* and *S. aureus*—via PCR, LFIA and the electrochemical method. One bacteria species using one detection method comprised 10 articles in this review. It is notable that the bacterial detection with the lowest LOD was selected in the current review when more than one bacteria species or detection method was investigated in the articles. It was difficult to keep track of detection efficiency, performance and LOD simultaneously. The multiplex detection capability was included as an important category in this review. The data were collected for bacteria species, year of article, multiplex detection capability, food sample type, sample number (number of samples tested for that bacteria species and food sample type in the article) and LOD (CFU/mL), shown in Table 1, Table 2 and Table 3. The sample number shows the repeatability of the experiment, which is important since it reflects successive measurements under the same conditions.

## 3. Results

**Figure 1:**
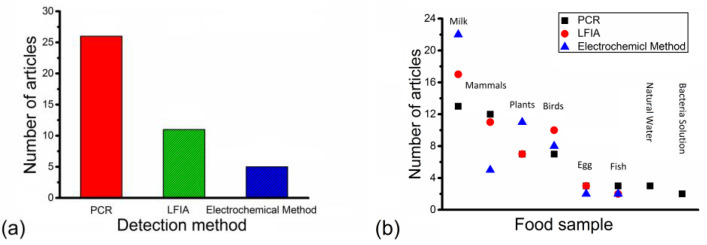
Number of articles (**a**) with multiplex detection capability and (**b**) food sample groups by different detection methods.

Table 1, Table 2 and Table 3 provide a breakdown of the analysis of the 150 peer-reviewed articles used in this review, based on the bacteria species, multiplex detection capability, food sample type, detection method and reported LOD. The food sample types were divided into eight groups for easier analysis: mammals (including beef, pork and sheep), birds (including chicken, duck, poultry and turkey), fish, egg, milk, plants (including lettuce, soybean, rice, cabbage and apple), natural water and bacterial solution (a solution that contains the target bacteria species, prepared via a conventional boiling method). The distribution of number of articles is further shown in Figure 1.

Figure 1a shows that the number of articles with multiplex detection capability was different among different detection methods. The number of articles with multiplex detection capability was the highest for PCR, followed by LFIA and the electrochemical method. The number of articles with multiplex detection capability for PCR, LFIA and the electrochemical method was 26 articles, 11 articles and 5 articles, respectively. Although most articles with multiplex detection capability only involved simultaneous detection of two bacteria species or two strains of one bacteria species, some articles with multiplex detection capability involved simultaneous detection of five bacteria species or five strains of one bacteria species (Table 1, Table 2 and Table 3). Figure 1b shows the numbers of articles according to food sample groups with varying detection methods. It was illustrated that milk was studied in the highest proportion of articles for each detection method. The second most-studied food sample group candidate was mammals via PCR and LFIA and plants via electrochemical method. The number of articles for milk for PCR, LFIA and the electrochemical method was 13 articles, 17 articles and 22 articles, respectively. The number of articles for mammals for PCR and LFIA was 12 articles and 11 articles, respectively. The number of articles for plants for the electrochemical method was 11 articles.

**Figure 2:**
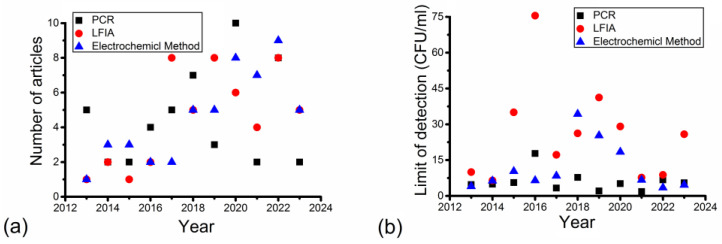
Timeline of the annual number of articles collected and average LODs in different years via different detection methods. (**a**) Number of articles. (**b**) Average LODs.

The distribution among the number of articles and the average LODs are annually presented in Figure 2. This figure shows the research trend of different detection methods in the years from 2013 to 2023 via different detection methods.

Figure 2a shows that the annual numbers of articles were different between different detection methods and years. There was at least one article published each year for each detection method from 2013 to 2023. The number of articles published from 2019 to 2023 was higher than that for articles published from 2013 to 2018 in each detection method, indicating increased research interest. For PCR, the annual number of articles was five articles in 2013; then, it decreased to two articles in 2014. It increased gradually to seven articles in 2018 and decreased to three articles in 2019. It reached its highest point at ten articles in 2020 and decreased to the bottom at two articles in 2021. In the case of LFIA, the annual number of articles was only one article in 2013. It increased gradually to eight articles in 2018. It reached eight articles again in 2019 and decreased to the lowest level at four articles in 2021. In the case of the electrochemical method, the annual number of articles also started with one article in 2013. It increased sharply to three articles in 2014 and decreased to two in 2016. Then, it increased gradually to nine in 2022, followed by a large decline to five in 2023.

Figure 2b shows that the average annual LOD was different between different detection methods and years. The annual average LOD was usually the highest for LFIA, except for the electrochemical method in 2018. The LOD was usually the lowest in PCR, except for the electrochemical method in 2016 and 2022. In PCR, the average LOD was 4 CFU/mL in 2013. Then, it increased gradually to 18 CFU/mL in 2016 and was followed by a large drop to 3 CFU/mL in 2017. After 2017, it decreased in an overall trend to 2 CFU/mL in 2021. It increased to 6 CFU/mL in 2022. In LFIA, it was 10 CFU/mL in 2013; it decreased to 7 CFU/mL in 2014 and increased tremendously to 75 CFU/mL in 2016. Then, it decreased sharply to 17 CFU/mL in 2017 and was again followed by an increase to 40 CFU/mL in 2019. After that, it decreased gradually to 8 CFU/mL in 2021 and increased again to 25 CFU/mL in 2023. In the electrochemical method, it was 4 CFU/mL in 2013, and it increased gradually to 10 CFU/mL in 2015. After a decrease to 7 CFU/mL in 2016, it increased again to 35 CFU/mL in 2018. Then, it decreased gradually to 3 CFU/mL in 2022.

**Figure 3:**
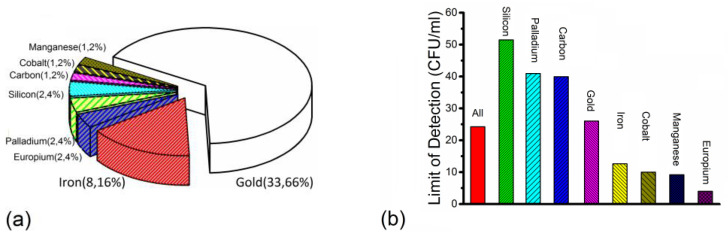
Bacterial detection via LFIA with different nanoparticles (white: gold; red: iron; dark blue: europium; green: palladium; light blue: silicon; pink: carbon; yellow: cobalt; brown: manganese). (**a**) Number of articles with different nanoparticles. (**b**) Average LOD with different nanoparticles.

Figure 3 shows the number of articles and average LODs for various nanoparticles from the reported articles for LFIA.

Figure 3a shows that the numbers of articles for LFIA were different with different nanoparticles. In all the articles for LFIA, gold was the most studied nanoparticle, followed by iron. The number of articles with gold or iron was 33 articles and 8 articles, respectively. Figure 3b illustrates that the average LODs were different among different nanoparticles. The average LOD was highest for articles with silicon, followed by articles with palladium and carbon. The average LOD of articles with silicon, palladium, carbon, gold, iron, cobalt, manganese and europium was 50 CFU/mL, 41 CFU/mL, 40 CFU/mL, 26 CFU/mL, 12 CFU/mL, 10 CFU/mL, 9 CFU/mL and 4 CFU/mL, respectively.

**Figure 4:**
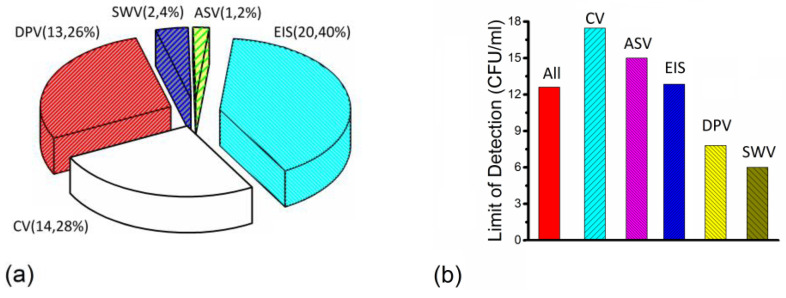
Bacterial detection via electrochemical method with different techniques (white: cyclic voltammetry (CV); red: differential pulse voltammetry (DPV); dark blue: square wave voltammetry (SWV); green: anodic stripping voltammetry (ASV); light blue: electrochemical impedance spectroscopy (EIS)). (**a**) Number of articles with different techniques. (**b**) Average LOD with different techniques.

The relationship between the number of articles and different techniques and average LOD is presented for the electrochemical method in Figure 4.

Figure 4a shows that the number of articles for the electrochemical method was different with different techniques. In all the articles for the electrochemical method, EIS was the most studied detection method, followed by CV and DPV. The number of articles with EIS, CV and DPV was 20 articles, 14 articles and 13 articles, respectively. Figure 4b illustrates that the average LODs via the electrochemical method were different between different techniques. The average LOD was the highest for articles with CV, followed by articles with ASV. The average LOD of articles with CV, ASV, EIS, DPV and SWV was 18 CFU/mL, 15 CFU/mL, 12 CFU/mL, 8 CFU/mL and 6 CFU/mL, respectively.

**Figure 5:**
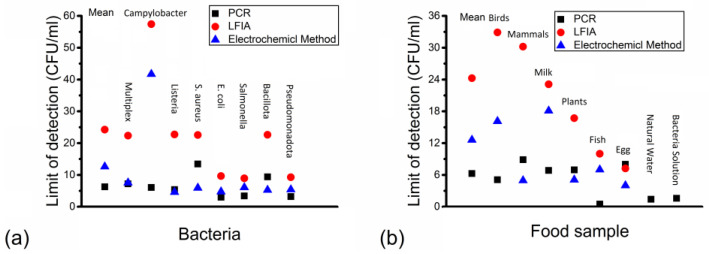
LODs of different bacteria species analyzed using different detection methods in the various food sample groups (in Table 1, Table 2 and Table 3). (**a**) LOD vs bacteria species. (**b**) LOD vs food sample groups.

Figure 5 shows the average LOD of different bacteria species and food sample groups via different detection methods based on 150 articles in tables. It shows which detection method is the most suitable for each bacteria species and food sample group.

Figure 5 presents the average LOD of different (a) bacteria species and (b)food sample groups via different detection methods. Figure 5a shows that the overall average LOD was the lowest for PCR and the highest for LFIA. The average LOD was higher for articles with multiplex detection capability in PCR than for those without, but it was lower in LFIA and the electrochemical method. PCR has the lowest average LOD among all detection methods for *Salmonella*, *Campylobacter* and *E. coli*, and these bacteria species are all gram-negative (−). In addition, the electrochemical method has the lowest average LOD among all detection methods for *Listeria* and *S. aureus*, and these bacteria species are all gram-positive (+). On the other hand, LFIA always has the highest average LOD for each bacteria species. In PCR and LFIA, the LODs for bacteria species in the Pseudomonadota domain were usually lower than those for bacteria species in the Bacillota, but they were similar to the latter for the electrochemical method. For bacteria species in the Pseudomonadota domain, the average LOD for *E. coli* was lower than it was for *Salmonella* in PCR and the electrochemical method, but higher than the latter in LFIA. For bacteria species in the Bacillota domain, the average LOD for *Listeria* was lower than it was for *S. aureus* in PCR and the electrochemical method, but higher than the latter in LFIA. The average LOD of *Campylobacter* was usually the highest among all the bacteria species in each detection method, except that it was lower than *S. aureus* in PCR.

Figure 5b shows that PCR had the lowest average LOD among birds, fish and milk, while the electrochemical method had the lowest average LOD among mammals, egg and plants. LFIA usually had the highest average LOD among all food sample groups, except that it was highest for PCR in egg. Among all the food sample groups, egg had the lowest average LOD for LFIA and the electrochemical method among all food sample groups, while fish had the lowest average LOD for PCR. In contrast, birds had the highest average LOD for PCR, which was followed by mammals and milk. Natural water and bacterial solution were only involved in detection for PCR, and their average LODs were lower than all other food sample groups except fish.

EU limits for *Salmonella*, *Listeria*, *Campylobacter*, *S. aureus* and *E. coli* in food for most people are 100, 100, 1000, 1000 and 100 CFU/mL, respectively [18]. All the LODs in this review are far lower than the EU limits stipulate. Although some foods intended for special groups, such as infants and sick people, require no presence of bacteria, at least one article with LOD within 0.3 CFU/mL was included in each bacteria species for PCR [18].

**Figure 6:**
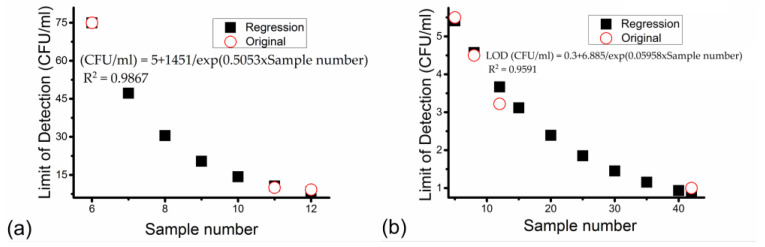
Exponential regressions of LODs of *Listeria* in milk via LFIA and electrochemical method with sample number. (**a**) Via LFIA. (**b**) Via electrochemical method.

In order to find an accurate quantitative relationship between LODs with different parameters, the LOD should be analyzed against each parameter individually. An exponential relation with relatively high Pearson correction coefficients (R^2^ > 0.95) can be obtained between the LOD of *Listeria* in milk via LFIA and the LOD of *Listeria* in milk via the electrochemical method with a sample number. The details of them can be seen in Figure 6, which shows both the LODs for the original and for the regression of *Listeria* in milk via LFIA and the electrochemical method.

For articles involving detection of *Listeria* in milk via LFIA, LOD (CFU/mL) = 5 + 1451/exp(0.5053 × sample number), R^2^ = 0.9867. For articles involving detection of *Listeria* in milk via the electrochemical method, LOD (CFU/mL) = 0.3 + 6.885/exp(0.05958 × sample number), R^2^ = 0.9591. These results show that the LOD usually decreases when the sample number increases for the detection of the same bacteria species and food sample group using the same detection method. However, the decreasing rate of LOD reduced gradually with an increasing sample number until LOD reached its lowest and remained unchanged after that. It can be seen that when the sample number is very large, the LOD of *Listeria* in milk via LFIA and the electrochemical method will be around 5 CFU/mL and 0.3 CFU/mL. This rule may also apply to other bacteria species and food sample groups, but more articles need to be collected and analyzed before a possible regression can be achieved.

## 4. Discussion

The review of the LODs for PCR, LFIA and the electrochemical method has revealed a trend in this research area that will inform food safety and public health experts. Figure 1a illustrates that the number of articles with multiplex detection capability is the highest in PCR, followed by LFIA and the electrochemical method. That is the main reason that PCR is considered a reliable standard detection method for bacterial detection under many circumstances. However, PCR does have the disadvantages of being a high-cost, time-consuming and complex procedure. As a result, PCR cannot replace LFIA and the electrochemical method for bacterial detection completely. Milk is the most popular food sample for bacterial detection via each detection method.

Figure 2 shows that although bacterial detection has attracted more attention from researchers in recent years, LODs in the published articles have not decreased continually. The main reason is that the LODs in this review are all lower than the EU limits.

Figure 3a shows that only three articles for LFIA involved non-metal nanoparticles (silicon: two, carbon: one). The majority of articles with LFIA involved metal nanoparticles. Figure 3b illustrates that average LODs for non-metal nanoparticles are usually higher than for metal nanoparticles, except that the average LOD of palladium is a little higher than that for carbon. In addition, four articles in detection of bacteria via LFIA involved combined detection (one article involved combined detection with the electrochemical method; three articles involved combined detection with PCR). For the same bacteria species, the LODs in articles involving combined detection with other methods were usually lower than the LODs in articles without. In the detection of *Salmonella,* the LOD in Ref. [101] was 1 CFU/mL, and it was lower than all LODs in other articles without combination with the electrochemical method. In the detection of *Listeria,* the LOD in Ref. [101] was 7 CFU/mL, and it was lower than all LODs in other articles without combination with PCR. In the detection of *S. aureus*, the LOD in Ref. [133] was 3 CFU/mL, and it was higher than the LODs in Ref. [131] and Ref. [132] without combination with PCR. However, the nanoparticle used in Ref. [133] was silicon while the nanoparticle used in the other two articles was gold, and the average LOD with silicon was higher than the LOD with gold. In addition, the LOD in Ref. [135] was 10 CFU/mL, and it was higher than the LODs in Ref. [131], Ref. [132] and Ref. [134] without combination with PCR. However, Ref. [125] involves the detection of five bacteria species simultaneously while the other three articles only involve the detection of *S. aureus*. It is very difficult to keep high detection efficiency and a low LOD simultaneously. These four articles combine the advantages of both detection methods, which can be a choice for further development of detection methods.

Figure 4 shows that few articles about the detection of *Campylobacter* with extremely high LODs (only *Campylobacter* includes articles with LODs over 20 CFU/mL via the electrochemical method) increase the average LODs via the electrochemical method.

In Figure 5a, the average LOD is the lowest for PCR in gram (−) bacteria species and for the electrochemical method in gram (+) bacteria species. *Campylobacter* is gram (−), and its average LOD is usually the highest among all bacteria species for each detection method, except that the LOD is the highest for *S. aureus* via PCR. A possible reason for the higher average LOD of *Campylobacter* and *S. aureus* is that EU limits for them are higher than for other bacteria species [18]. The average LODs for *Salmonella* and *E. coli* (both gram (−)) in the Pseudomonadota domain are usually lower than those for *Listeria* and *S. aureus* (both gram (+)) in the Bacillota domain in PCR and LFIA, but similar to the latter in the electrochemical method. The difference between two bacteria species in the same domain is much smaller than the difference between different domains.

Furthermore, the sample number plays an important role in controlling the LOD in each method. Figure 6 shows that the exponential formulas fulfil the original data of LODs of *Listeria* in milk via LFIA and the electrochemical method with sample numbers from already published research articles. These exponential regressions involve the LOD of *Listeria* and sample numbers in LFIA and the electrochemical method in milk samples. The main reason is that milk is the most common food sample in each detection method in this review, and its composition is simpler than that of meat samples [201].

This review also shows that the average LOD for articles with multiplex detection capability is higher than for articles without in PCR, but lower in LFIA and the electrochemical method. One of the possible reasons is that PCR usually has a lower LOD than LFIA and the electrochemical method. It is difficult to keep both detection efficiency and sensitivity at the same time when LOD is already low. This could be a promising focus for the development of bacterial detection in the future. This review also indicates that fish and egg have the lowest average LOD among all food sample groups. The complexity of the food sample composition can increase the LOD. To address such limitations and challenges, sample enrichment and improvement in the device properties of detection are needed. PCR, LFIA and the electrochemical method have been used in detection of different bacteria species, and many of them involve multiplex detection. It is often observed that bacteria species coexist in a single food sample. As a result, multiplex detection is needed that can fulfill the requirements of a low LOD and high efficiency simultaneously. These detection methods can also be combined with other technologies to obtain a better detection performance.

### Challenges and Future Perspectives

Sensitivity and Specificity: Enhancing sensitivity and specificity poses a significant challenge. The integration of specific aptamers or DNA strands enhances PCR-based bacterial detection in terms of sensitivity and specificity. For LFIA, lateral-flow design and integration of monoclonal antibodies and nanomaterials seem crucial for enhancing specificity and LODs. For the electrochemical method, electrode modification with diverse nanomaterials has emerged as a prevalent technique, amplifying signals and improving sensitivity. MALDI-TOF mass spectrometry is a widely used technique in electrochemical methods for the bacterial detection to increase reliability, accuracy and efficiency. Microfluidic platforms offer a seamless integration with LFIA and the electrochemical method. The colorimetric and fluorescent sensing methods can be used in PCR, LFIA and electrochemical methods to achieve lower LODs and wider linear ranges. In addition, all PCR, LFIA and electrochemical methods can be used in bacteria drug resistance tests [202,203].

Sample Complexity: Addressing the challenges related to sample complexity and matrix effects and cost is crucial for the development of efficient bacterial detection systems. Sample complexity can lead to a higher LOD, and LOD is also affected by pretreatment of food samples. As a result, complex biosensing systems necessitate pretreatment of food samples, with different food samples requiring varied sample treatments and techniques. Achieving data under similar sample treatments and identical testing conditions is challenging but important.

Analysis Time: The total time required for analysis varies across different bacterial detection methods, including PCR, LFIA and electrochemical methods. LFIA and electrochemical methods are well known for their rapid analysis and multiplex detection capability.

Role of nanomaterials and advanced materials for future developments: The integration and successful utilization of various materials and nanomaterials for bacterial detection in food is well reported in recent years. Nanomaterials offer unique properties, including high surface area, tunable physical characteristics and enhanced reactivity, which makes them ideal candidates for improving sensitivity, specificity and overall performance.

PCR: Nanomaterials find a major application in PCR-based bacterial detection methods, contributing to the sensitivity and efficiency of the amplification process. Nanoparticles such as AuNPs, silicon and magnetic nanoparticles are often utilized in PCR assays. One significant application is in the extraction/purification of nucleic acids from bacterial samples. Magnetic nanoparticles coated with specific ligands can bind to bacterial DNA or RNA selectively, enabling the isolation from food matrices. This enhances purity and subsequently improves the reliability of PCR amplification. Additionally, nanoparticles, as labels for detection, can help in facilitating the visualization of PCR products. Quantum dots, for instance, provide a fluorescent signal which can be quantified, enhancing the sensitivity and specificity of bacterial detection via PCR [204].

LFIA: Nanomaterials play a crucial role in enhancing the performance of LFIA for bacterial detection in food. Carbon nano-tubes, magnetic nanoparticles and quantum dots are among the commonly utilized nanomaterials. These materials are employed for conjugation with antibodies specifically related to the targeted species. Nanomaterials are normally integrated into the test strip, e.g., AuNPs are frequently utilized as labels for bacterial detection (due to their distinct color change properties). The immobilization of antibodies on the surface of these nanoparticles facilitates specific binding to bacterial antigens, thereby enabling the quantitative detection of the target bacteria species. Moreover, the use of nanomaterials in LFIA is reported to help in signal amplification and improved sensitivity (and lower LOD) [205].

Electrochemical method: Nanomaterials play a crucial role in enhancing the performance of electrochemical methods for bacterial detection. Carbon-based nanomaterials, metal nanoparticles and nanocomposites are commonly integrated onto the electrode surfaces to improve the response and signal amplification. Nanomaterials provide an improved surface area for the immobilization of specific recognition elements (antibodies or aptamers), which ensures efficient capture of the target bacteria species, thereby improving sensitivity. In addition, nanomaterials modify the electrode surface to promote electron transfer kinetics and hence result in rapid and reliable electrochemical signals and detection. The unique properties of nanomaterials, such as size, structure, conductivity and catalytic activity, contribute to the overall performance of electrochemical biosensors for bacterial detection [206,207,208,209]. To make a comparison of PCR, LFIA and electrochemical methods for bacterial detection, Table 4 is listed below.

In summary, the integration of nanomaterials in PCR, LFIA and electrochemical methods for bacterial detection in food represents a promising strategy to overcome the challenges associated with sensitivity, specificity, overall performance and LODs. The exploration of novel nanomaterials and their tailored applications would help us to further lower the LODs and advance the capability of bacterial detection in food safety.

## 5. Conclusions

The development of detection technology for monitoring the quality and safety of foods has provided promising tools for improved quantitative performance. In order to improve the accuracy and precision of different detection methods (PCR, LFIA and electrochemical method), different parameters such as bacteria species, year of article, multiplex detection capability and food sample type have been considered as determinants of LOD. The results show that bacteria species and food sample type strongly contribute to predicting the LOD. Average LOD is the highest for detection using LFIA (24 CFU/mL), followed by the electrochemical method (12 CFU/mL) and PCR (6 CFU/mL). *Salmonella* and *Escherichia coli* in the Pseudomonadota domain usually have lower LODs than other bacteria species. LODs are usually lower for detections in fish and egg than for detections in other food samples analyzed. Most articles about LFIA involve metal nanoparticles—especially gold and iron. The average LOD of articles involving gold (26 CFU/mL) is higher than that of iron (12 CFU/mL). EIS, CV and DPV are three major techniques among articles about electrochemical methods. CV has a higher average LOD (18 CFU/mL) than EIS (12 CFU/mL) and DPV (8 CFU/mL). The LOD usually decreases when the sample number increases until it reaches its lowest point in the detection of the same bacteria species, food sample group and detection method. The LODs of *Listeria* in milk using LFIA and an electrochemical method with sample numbers have exponential regressions with relatively high Pearson correction coefficients (R^2^ > 0.95). Sample enrichment and improvement in device properties of detection and the possibility of combination with other detection technologies are needed to lower the LOD and improve the performance of detection further. This review provides guidance for future developments in bacteria monitoring technologies based on the enrichment of bacteria from samples and the development of multiplex detection methods that can increase the detection efficiency but also keep the LOD low. The integration and exploration of novel nanomaterials will help to further lower the LOD and advance the capability of bacterial detection technologies in the realm of food safety.

## 6. Methods

**Figure 7:**
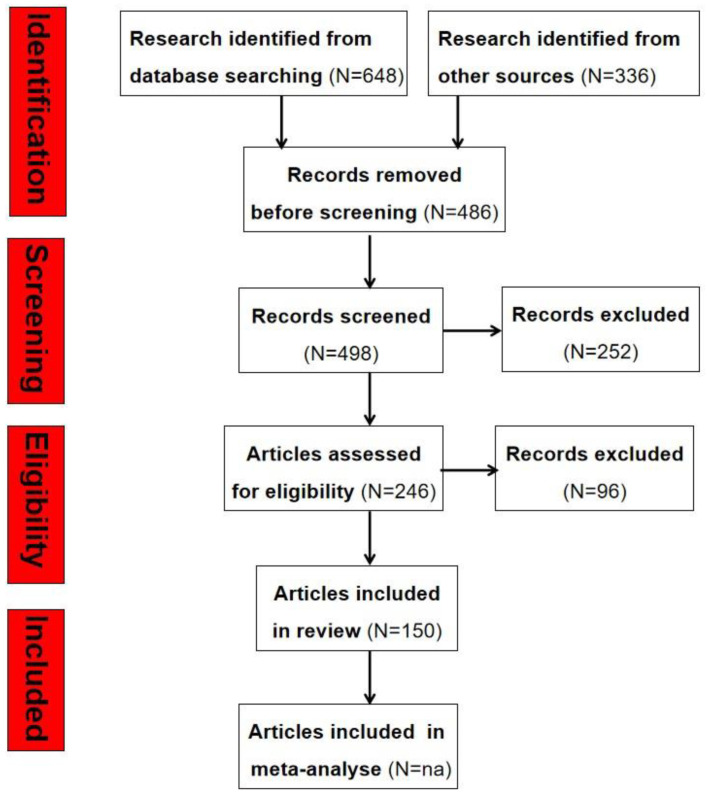
PRISMA flow diagram for the literature search; na = not applicable.

PRISMA Statement (Preferred Reporting Items for Systematic Reviews and Meta-Analyses): We finished the PRISMA 2020 checklist and constructed a flowchart following the PRISMA guidelines and registration information. The selection process was based on the PRISMA statement 2020 [210], and the flowchart is shown in Figure 7.

Research Process: Most foodborne diseases are caused by bacteria in food, and they can be infectious and dangerous. It is essential to detect bacteria in food quickly and accurately. The systematic review was gathered through a literature search from online databases. Relevant articles were searched on Google Scholar and the Scopus database to identify the LODs of common detection methods—PCR, LFIA and electrochemical methods—in bacterial detection in food. The Boolean operators “AND” and “OR” were used to broaden the search. The keywords used for searching were “LOD”, “*Salmonella*”, “*Listeria*”, “*Campylobacter*”, “*S. aureus*”, “*E. coli*”, “PCR”, “LFIA” and “electrochemical method”. The article was identified through the Scopus database and Google Scholar online. The citations were collected from recent studies (2013–2023). To further ensure that we had assembled a comprehensive list of studies, we asked researchers with relevant knowledge on the topic to review and suggest keywords. The search focused on scientific research articles using the following protocol:

i. Publication years were between 2013 and 2023.
ii. The keywords “(“LOD”)” AND “(“*Salmonella*” OR “*Listeria*” OR “*Campylobacter*” OR “*S. aureus*” OR “*E. coli*”)” AND “(“PCR” OR “LFIA” OR “electrochemical method”)” had to appear in the title and/or abstract.
iii. They had to be scientific indexed papers with lowest LODs only.

The results were screened against inclusion criteria, i.e., articles that were not relevant to the studies. The full text of papers for all the articles that fit into the inclusion criteria was retrieved.

Screening: Strict criteria were used to determine the relevant articles for inclusion. For example, articles were excluded if published in languages other than English or for which only an abstract was available, and then each remaining search result was grouped as one of the articles.

i. “Primary articles” were research papers that appeared in the peer-reviewed literature and reported original data or results based on observations and experiments.
ii. “Review” papers summarized the understanding of the LODs of five bacteria species using three detection methods.

Throughout the screening process, the number of publications excluded in each stage and their reasons for exclusion were noted based on the guidelines outlined in the PRISMA statement 2020 in Figure 7.

## Figures and Tables

**Table 1:** PCR: Papers with LODs for five common bacteria species.

Bacteria	Multiplex Detection Capability	Food Sample	Sample Number	LOD (CFU/mL)	Year	Reference
*Salmonella*	*No*	Beef	60	0.04	2022	[51]
	*No*	Chicken	10	0.1	2017	[52]
	*Salmonella* + *Listeria*	Bacterial Solution	8	0.2	2013	[53]
	Two *Salmonella* strains	Pork	7	2	2019	[54]
	*No*	Lettuce	18	2.65	2021	[55]
	*Salmonella* + *Listeria*	Bacterial Solution	6	3	2022	[56]
	*Salmonella* + *Pseudomonas* + *Bacillus*	Natural Water	8	3	2020	[57]
	Two *Salmonella* strains	Chicken	6	4	2018	[58]
	*No*	Sheep	7	9	2020	[59]
	*No*	Chicken	6	10	2017	[60]
*Listeria*	Two *Listeria* strains	Fish	9	0.2	2022	[61]
	*Listeria* + *Salmonella* + *S. aureus*	Egg	50	0.2	2014	[62]
	*Listeria* + *Salmonella* + *E. coli*	Duck	160	0.48	2022	[63]
	*No*	Soybean	20	4	2019	[64]
	*No*	Milk	35	5	2017	[65]
	*No*	Milk	6	5	2022	[66]
	*Listeria* + *Salmonella* + *E. coli* + *Shigella* + *Yersinia*	Pork	5	9	2013	[67]
	*Listeria* + *Brucella*	Milk	13	10	2023	[68]
	Two *Listeria* strains	Lettuce	14	10	2022	[69]
	Two *Listeria* strains	Lettuce	21	10	2016	[70]
*Campylobacter*	*No*	Pork	8	0.3	2014	[71]
	*No*	Milk	5	1	2023	[72]
	Five *Campylobacter* strains	Milk	8	1	2020	[73]
	Two *Campylobacter* strains	Chicken	9	1	2017	[74]
	*No*	Sheep	41	4.3	2013	[75]
	*No*	Pork	30	10	2013	[76]
	*No*	Pork	54	10	2020	[77]
	*No*	Chicken	40	10	2018	[78]
	*No*	Chicken	6	10	2013	[79]
	*No*	Milk	12	13	2020	[80]
*S. aureus*	*No*	Milk	24	0.25	2019	[81]
	*S. aureus* + *Salmonella* + *Listeria*	Milk	46	0.48	2017	[82]
	*No*	Fish	150	1.2	2018	[83]
	*No*	Egg	50	3.8	2020	[84]
	*S. aureus* + *Salmonella* + *Shigella*	Pork	51	9.6	2014	[85]
	Five *S. aureus* strains	Milk	13	10	2022	[86]
	*S. aureus* + *Bacillus* + *Cronobacter*	Rice	8	19	2016	[87]
	*S. aureus* + *Salmonella* + *Listeria*	Egg	12	20	2022	[88]
	*S. aureus* + *Enterobacter* + *Proteus*	Milk	5	28	2018	[89]
	*S. aureus* + *Salmonella* + *Listeria* + *E. coli* + *Shigella*	Beef	9	42	2016	[90]
*E. coli*	*No*	Natural Water	6	0.04	2018	[91]
	Four *E. coli* strains	Fish	180	0.12	2016	[92]
	Two *E. coli* strains	Beef	32	0.14	2020	[93]
	*E. coli* + *Salmonella*	Cabbage	25	1	2018	[94]
	*No*	Milk	10	1.03	2021	[95]
	*No*	Natural Water	7	1.2	2015	[96]
	Three *E. coli* strains	Apple	22	2	2020	[97]
	*No*	Milk	7	4.4	2020	[98]
	*No*	Beef	12	10	2018	[99]
	*E. coli* + *Listeria*	Milk	8	10	2015	[100]

Notes: Multiplex detection capability: whether the article involves detection of two or more bacteria species or two or more strains of one bacteria species simultaneously. Sample number: number of samples tested for that bacteria species and food sample type in the article.

**Table 2:** **LFIA:** Papers with LODs for five common bacteria species.

Bacteria	Multiplex Detection Capability	Combined Method	Food Sample	Sample Number	Nanoparticle	LOD (CFU/mL)	Year	Reference
*Salmonella*	*No*	Dual colorimetric/electrochemical immunosensors, based on antibody	Orange	8	Gold	1	2023	[101]
	*No*		Chicken	5	Gold	1	2019	[102]
	*No*	*No*	Chicken	6	Gold	1	2018	[103]
	*No*	*No*	Egg	11	Gold	1.05	2017	[104]
	*No*	*No*	Milk	7	Gold	1.6	2017	[105]
	2 types of *Salmonella*	*No*	Grape	9	Iron	8	2022	[106]
	*No*	*No*	Milk	7	Gold	8.6	2021	[107]
	*No*	*No*	Chicken	5	Iron	16	2019	[108]
	*No*	*No*	Lettuce	6	Gold	17	2023	[109]
	*No*	*No*	Milk	5	Iron	34	2019	[110]
*Listeria*	*Listeria + E. coli* + *Vibrio*	Europium-based fluorescent LFIA + PCR, based on nucleic acid	Beef	6	Europium	7	2021	[111]
	*No*	*No*	Pork	30	Gold	8	2023	[112]
	*No*	*No*	Milk	12	Manganese	9.2	2021	[113]
	*No*	*No*	Lettuce	5	Iron	10	2022	[114]
	*No*	*No*	Milk	11	Gold	10	2017	[115]
	*No*	*No*	Pork	6	Gold	11	2022	[116]
	*Listeria* + *Salmonella*	*No*	Egg	9	Gold	19	2017	[117]
	*No*	*No*	Lettuce	6	Gold	30	2017	[118]
	*No*	*No*	Lettuce	5	Palladium	48	2020	[119]
	*Listeria* + *Salmonella*	*No*	Milk	6	Gold	75	2019	[120]
*Campylobacter*	*No*	*No*	Milk	7	Iron	3	2022	[121]
	*No*	*No*	Poultry	60	Gold	10	2018	[122]
	*Campylobacter* + *Salmonella* + *S. aureus*	*No*	Poultry	9	Iron	10	2018	[123]
	*Campylobacter* + *Salmonella* + *S. aureus*	*No*	Poultry	8	Cobalt	10	2018	[124]
	*No*	*No*	Fish	105	Iron	10	2014	[125]
	*No*	*No*	Milk	6	Gold	50	2019	[126]
	*No*	*No*	Chicken	6	Gold	100	2020	[127]
	*No*	*No*	Pork	112	Gold	100	2018	[128]
	*No*	*No*	Chicken	7	Gold	131	2019	[129]
	*No*	*No*	Sheep	5	Gold	150	2016	[130]
*S. aureus*	*No*	*No*	Egg	6	Gold	1.6	2022	[131]
	*No*	*No*	Pork	9	Gold	2	2017	[132]
	*No*	Quantum dot-based LFIA + double labeling PCR, based on antibody	Milk	7	Silicon	3	2014	[133]
	*No*	*No*	Sheep	36	Gold	5.96	2021	[134]
	*S. aureus* + *Salmonella* + *Listeria* + *E. coli* + *Vibrio*	LFIA+PCR with automatic nucleic acid extractor, based on nucleic acid	Fish	8	Gold	10	2022	[135]
	*No*	*No*	Milk	30	Gold	10	2013	[136]
	2 *S. aureus* strains	*No*	Milk	32	Gold	18	2023	[137]
	2 *S. aureus* strains	*No*	Beef	6	Gold	35	2015	[138]
	*No*	*No*	Turkey	6	Carbon	40	2017	[139]
	*No*	*No*	Milk	6	Silicon	100	2023	[140]
*E. coli*	*No*	*No*	Pork	50	Europium	1	2020	[141]
	*No*	*No*	Milk	7	Gold	1	2016	[142]
	*No*	*No*	Pork	8	Gold	2.2	2023	[143]
	*No*	*No*	Milk	5	Gold	2.7	2019	[144]
	*No*	*No*	Apple	7	Gold	3	2020	[145]
	*No*	*No*	Chicken	7	Iron	10	2022	[146]
	*No*	*No*	Beef	10	Gold	10	2020	[147]
	*No*	*No*	Milk	5	Gold	12.5	2020	[148]
	2 *E. coli* strains	*No*	Milk	6	Gold	20	2019	[149]
	*E. coli* + *Salmonella*	*No*	Milk	8	Palladium	34	2017	[150]

Notes: Combined methods: whether the article involves detection of the same bacteria species with detection methods besides LFIA.

**Table 3:** Electrochemical method: papers with LODs for five common bacteria species.

Bacteria	Multiplex Detection Capability	Food Sample	Sample Number	Electrochemical Technique	LOD (CFU/mL)	Year	Reference
*Salmonella*	Two *Salmonella* strains	Milk	8	DPV	2.6	2022	[151]
	*No*	Apple	7	EIS	3	2016	[152]
	*No*	Pork	8	CV	3	2014	[153]
	*No*	Egg	10	EIS	5	2020	[154]
	*No*	Milk	5	CV	5	2015	[155]
	*No*	Milk	5	DPV	6	2021	[156]
	*No*	Milk	9	EIS	6	2014	[157]
	*No*	Chicken	8	DPV	10	2020	[158]
	*No*	Chicken	7	DPV	10	2019	[159]
	*No*	Apple	8	EIS	10	2016	[160]
*Listeria*	*No*	Milk	42	SWV	1	2023	[161]
	*No*	Lettuce	12	CV	2	2023	[162]
	Two *Listeria* strains	Milk	12	EIS	3.22	2021	[163]
	*No*	Pork	6	EIS	4	2020	[164]
	*No*	Tomato	6	EIS	4	2013	[165]
	*No*	Milk	8	EIS	4.5	2022	[166]
	*No*	Chicken	6	CV	5	2022	[167]
	*No*	Milk	5	EIS	5.5	2018	[168]
	*No*	Pork	25	DPV	6.8	2022	[169]
	*No*	Lettuce	5	DPV	10	2021	[170]
*Campylobacter*	*No*	Beef	31	EIS	8	2023	[171]
	*No*	Poultry	118	EIS	10	2021	[172]
	*No*	Chicken	156	DPV	10	2020	[173]
	*No*	Poultry	100	SWV	11	2015	[174]
	*No*	Chicken	36	DPV	13	2018	[175]
	Two *Campylobacter* strains	Poultry	7	ASV	15	2015	[176]
	*No*	Chicken	50	EIS	50	2019	[177]
	*No*	Milk	6	EIS	100	2020	[178]
	*No*	Milk	5	CV	100	2020	[179]
	*No*	Milk	5	CV	100	2019	[180]
*S. aureus*	*No*	Apple	9	CV	1	2022	[181]
	*No*	Apple	7	CV	1	2022	[182]
	*No*	Milk	6	CV	2	2022	[183]
	*No*	Pork	7	EIS	3	2021	[184]
	*No*	Milk	7	EIS	3.3	2020	[185]
	*No*	Orange	9	CV	5	2022	[186]
	*No*	Milk	6	DPV	5	2020	[187]
	*No*	Fish	7	EIS	10	2014	[188]
	*No*	Milk	7	DPV	13	2017	[189]
	*S. aureus* + *Salmonella*	Milk	7	EIS	15.9	2021	[190]
*E. coli*	*No*	Milk	7	DPV	2	2021	[191]
	*No*	Egg	6	CV	3	2022	[192]
	*No*	Milk	6	DPV	3	2019	[193]
	*No*	Milk	8	EIS	3	2018	[194]
	*No*	Milk	5	CV	3.5	2019	[195]
	*No*	Milk	9	EIS	3.8	2017	[196]
	*No*	Fish	5	CV	4	2022	[197]
	*E. coli* + *Salmonella*	Lettuce	12	EIS	5	2023	[198]
	*No*	Apple	9	CV	10	2020	[199]
	*No*	Milk	5	DPV	10	2019	[200]

Notes: Electrochemical techniques: EIS: electrochemical impedance spectroscopy; CV: cyclic voltammetry; DPV: differential pulse voltammetry; SWV: square wave voltammetry; ASV: anodic stripping voltammetry.

**Table 4:** Comparison of PCR, LFIA and electrochemical methods for bacterial detection.

Detection Method	Principle	LOD (CFU/mL)	Analysis Time	Sample Preparation	Matrix Effect	Analysis Complexity
PCR	PCR amplifies a specific region of a DNA strand to make many copies of a DNA strand.	0.1–10	3–18 h	Collects the bacteria, removes the inhibitors in the food sample, concentrates template for PCR.	Most by PCR inhibition, disturbs detection, false negative.	Highest, complex
LFIA	Liquid sample moves through a polymeric strip, with attached molecules interacting with the targeted bacteria.	1–1000	3–15 min	Food sample is mixed with buffered water and diluted. Then, diluents are collected and separated.	Most by sample complexity, steps of sample collection, etc.	Lowest, complex
Electrochemical method	Bacteria in the liquid result in changes in electrochemical signals.	1–100	15–60 min	Similar to LFIA, varies between different technologies.	Most by sample reactions with bacteria sensor, matrix, etc.	Low, complex
